# The effects of deoxynivalenol-contaminated corn in low-complexity diets supplemented with either an immune-modulating feed additive, or fish oil on nursery pig growth performance, immune response, small intestinal morphology, and component digestibility

**DOI:** 10.1093/tas/txac068

**Published:** 2022-05-19

**Authors:** Élise Lafleur Larivière, Cuilan Zhu, Ankita Sharma, Niel A Karrow, Lee-Anne Huber

**Affiliations:** Department of Animal Biosciences, University of Guelph, Guelph, ON, N1G 2W1, Canada; Department of Animal Biosciences, University of Guelph, Guelph, ON, N1G 2W1, Canada; Department of Animal Biosciences, University of Guelph, Guelph, ON, N1G 2W1, Canada; Department of Animal Biosciences, University of Guelph, Guelph, ON, N1G 2W1, Canada; Department of Animal Biosciences, University of Guelph, Guelph, ON, N1G 2W1, Canada

**Keywords:** deoxynivalenol, growth performance, immune response, low-complexity diets, nursery pigs

## Abstract

Three hundred twenty newly weaned pigs (21 days of age; 6.7 ± 0.3 kg BW) were used to determine the effects of supplementing low-complexity (**LC**) deoxynivalenol- (**DON**) contaminated nursery diets with a feed additive or fish oil on growth performance and immune response to an *Escherichia coli* lipopolysaccharide (**LPS**) challenge. Pens were randomly assigned to 1 of 5 dietary treatments (*n* = 8 pens per treatment): positive control (**PC**; contained multiple animal protein sources), or 1 of 4 LC diets (contained only plant-based protein sources) without (**NC**; negative control) or with ~ 3.5 ppm DON contamination, without (**DON‐**) or with a feed additive containing a blend of immune-modulating components (**DON+**; 2 mg/kg, as-fed) or fish oil (**DONω3**; 2.5%, as-fed). Dietary treatments were fed during phases I and II (7 and 15 days, respectively) and a common phase III diet was fed for 20 days. On day 22, two pigs per pen were injected IM with 30 μg/kg BW LPS and 1 pig per pen with 1 mL saline. Rectal temperatures were recorded at 0, 1, 2, 3 h after injection. At 3 h, blood was collected for plasma cytokine analysis and small intestinal histomorphology was assessed. In phase I, pigs fed PC and NC did not differ for ADG, ADFI and G:F, but these outcomes were greater than for pigs fed DON+ and DONω (*P* < 0.05). In phase II, pigs fed NC had greater ADG and PC had greater ADFI but lower G:F than pigs fed DON‐ and DONω3 (*P* < 0.05). At the end of phase II, pigs fed DONω3 tended to have lower BW than PC and NC (*P* = 0.084 and 0.079, respectively). In phase III and overall, there were no differences among dietary treatments for ADG, ADFI, G:F, or final BW. The LPS injection increased rectal temperature and reduced jejunal and ileal villus height (versus saline; *P* < 0.05). Plasma interferon-γ concentration was only increased by LPS for pigs fed PC, NC, and DON+ compared to the saline-injected counterparts (*P* < 0.05). Regardless of LPS injection, jejunal villus height was greater for pigs fed DON+ than DONω3 (*P* < 0.05) and ileal villus height was greater for pigs fed DON+ and PC than DONω3 (*P* < 0.05). Therefore, nursery diet complexity did not affect growth performance or immune response to LPS. Regardless of DON contamination and feed additive inclusion in phases I and II, pigs were able to achieve nursery exit BW not different from those fed PC. The feed additive offered marginal benefits for small intestinal villus height and immune response for pigs fed DON-contaminated LC nursery diets.

## INTRODUCTION

Exposure to novel pathogens and food allergens, an immature gastrointestinal tract, and other environmental stressors are factors that contribute to the marked reduction in feed intake and the resulting post-weaning growth lag for pigs (Lallès et al., 2004). In an attempt to attenuate this loss in growth performance, nursery diets commonly contain multiple sources of highly digestible animal-based proteins (Campbell and Dunkin, 1983; Koo et al., 2020). Previous research has demonstrated however, that pigs fed nursery diets with lower quality protein sources (e.g., soybean meal; **SBM**) can have accelerated growth after an initial reduction in ADG and achieve the same BW as those fed high quality protein sources immediately after weaning, referred to as compensatory growth (Skinner et al., 2014; Huber et al., 2018).

In addition to containing allergenic proteins (Li et al., 1991; Chen et al., 2011) and poorer amino acid digestibility versus diets containing animal-based protein sources (Cervantes-Pahm and Stein, 2010), simple, plant-based nursery diets including SBM also have higher inclusion rates of cereal grains that may be contaminated with mycotoxins. In North America, deoxynivalenol-(**DON**) producing *Fusarium* fungi are among the most prevalent to infest cereal grains (Pestka, 2007; Rodrigues and Naehrer, 2012). Moreover, at levels greater than 3 ppm, DON reduces feed intake and ADG and alters immune function (Pestka and Smolinksi, 2005), which could interfere with a pig’s ability to achieve compensatory growth when fed simple, plant-based diets (Larivière et al., 2021). Conversely, certain immune-modulating ingredients have been linked to reduced oxidative stress (vitamin E; Frankič et al., 2008), reduced inflammation (omega-3 fatty acids; Duan et al., 2014), and improved growth performance (preservation compounds, antioxidants, and amino acid blend; Van Le Thanh et al., 2015), which could help to alleviate the negative impacts of DON contamination on the pig’s ability to achieve compensatory growth.

Therefore, the objectives of the current study were to evaluate the effects of DON-contaminated low-complexity nursery diets containing either an immune-modulating feed additive (i.e. containing a blend of vitamins, yeast autolysate, and inorganic absorbent) or fish oil on growth performance, immune response to an *Escherichia coli* lipopolysaccharide (**LPS**) challenge, gut morphology, and component digestibility.

## MATERIALS AND METHODS

The experimental protocol was approved by the University of Guelph Animal Care Committee (AUP #4404) and followed Canadian Council of Animal Care guidelines (CCAC, 2009). The study was conducted at the Arkell Swine Research Station at the University of Guelph (Guelph, ON, Canada).

### Animals and Experimental Diets

Three hundred twenty Yorkshire × Landrace × Duroc pigs (160 castrated males and 160 females) were weaned at 21 ± 2.1 days of age in two weaning batches (blocks; 6.7 ± 0.3 kg BW). At weaning, the pigs were divided into 40 pens, with 8 pigs per pen (4 castrated males and 4 females; pens were balanced for BW and littermates were assigned to different pens). Pens were assigned to one of five dietary treatments in a randomized complete block design (*n* = 8 pens per dietary treatment; study day 0). Dietary treatments were fed over two phases (i.e. treatment phase; phases I and II were fed for 7 and 15 days, respectively). During phase III, a common diet was fed to all pens for an additional 20 days (i.e. recovery phase). Phase I diets were crumbled and phase II and III diets were pelleted. Phase II diets contained titanium dioxide to determine apparent total tract digestibility (**ATTD**) of components. Diets were formulated to meet or exceed estimated nutrient requirements for nursery pigs (NRC, 2012; Table 1). Pigs had ad libitum access to feed via a four-space feeder and to water via a nipple drinker in each pen. Individual pig BW and per pen feed disappearance were recorded weekly to determine ADG, ADFI, and G:F in each phase.

**Table 1.** Ingredient and calculated nutrient composition of experimental diets (as-fed basis)^1^

Item	PC		LC				PIII
			NC/DON- /DON+^2^		DONω3		
	PI	PII	PI	PII	PI	PII	
Ingredient, %							
Corn	47.59	58.49	55.12	52.88	55.12	52.88	59.75
Soybean meal dehulled	10.00	15.00	35.00	37.32	35.00	37.32	31.22
Soy protein isolate^3^	9.00	3.00	–	–	–	–	–
Whey, dried	20.00	10.00	–	–	–	–	–
Blood meal	–	2.00	–	–	–	–	–
Blood plasma^4^	5.00	2.00	–	–	–	–	–
Corn oil^5^	5.00	5.00	5.00	5.00	2.50	2.50	5.00
Fish oil^6^	–	–	–	–	2.50	2.50	–
L-lysine-HCl	0.20	0.50	0.50	0.41	0.50	0.41	0.40
DL-methionine	0.13	0.20	0.19	0.17	0.19	0.17	0.14
L-threonine	–	0.14	0.13	0.12	0.13	0.12	0.12
Limestone	1.36	1.33	1.30	1.33	1.30	1.33	1.21
Salt	–	0.33	0.60	0.76	0.60	0.76	0.64
Monocalcium phosphate	1.10	1.19	1.54	1.19	1.54	1.19	0.90
Vitamin and mineral premix^7^	0.60	0.60	0.60	0.60	0.60	0.60	0.60
Vitamin E^8^	0.02	0.02	0.02	0.02	0.02	0.02	0.02
Titanium oxide	–	0.20	–	0.20	–	0.20	–
Calculated nutrient composition^9^							
NE, Kcal/kg	2734	2721	2610	2600	2609	2599	2650
Crude protein, %	22.81	19.72	21.94	22.76	21.94	22.78	20.38
SID^10^ Lys, %	1.42	1.42	1.43	1.42	1.43	1.42	1.26
SID Met, %	0.41	0.45	0.48	0.47	0.48	0.47	0.41
SID Met + Cys, %	0.77	0.74	0.77	0.77	0.77	0.77	0.69
SID Thr, %	0.80	0.79	0.80	0.82	0.80	0.83	0.74
Calcium, %	0.95	0.93	0.97	0.93	0.97	0.93	0.82
Total P, %	0.75	0.69	0.78	0.71	0.78	0.71	0.62
STTD^11^ P, %	0.55	0.48	0.50	0.44	0.50	0.44	0.37
ω6:ω3	20.78	22.0	19.2	18.7	2.2	2.2	20.0

Experimental diets: [1] complex diet containing multiple sources of plant and animal proteins (positive control; PC), [2] low-complexity (LC) diets containing plant proteins as the main sources of protein, using corn with minimal deoxynivalenol content (negative control; NC), or corn with high DON contents and no feed additive (DON–), or with the feed additive to replace corn (at 0.2%; DON+), or supplemented with fish oil (DONω3). Phases I and II were fed between days 0 and 7 and 8 and 22 after weaning (treatment phases), respectively, and a common phase III was fed to all pigs between days 23 and 42 after weaning (recovery phase).

NutraMix™ (Canadian Bio-Systems Inc., Calgary, AB, Canada); contained per kilogram, min. 39,650 I.U. vitamin D3, min. 2,600 I.U. vitamin E, min. 1,900 mg niacin, min. 440 mg thiamine, min. 330 mg riboflavin, min. 1,000 mg calcium d-pantothenate, 220 mg pyridoxine, 1,000 µg biotin, 2,000 µg vitamin B12, min. 80 mg menadione, dehydrated yeast autolysate, and montmorillonite clay; included in DON+ diets.

Ardex^®^ AF; Manufactured by Archer Daniels Midland company (Decatur, IL).

AP920; manufactured by APC Nutrition Inc. (Ames, IA).

Corn oil, Saporito Foods Inc. (Markham, ON, Canada).

Menhaden fish oil; Grand Valley Fortifiers (Cambridge, ON, Canada).

Provided, per kilogram of diet, 12,000 IU vitamin A as retinyl acetate, 1,200 IU vitamin D3 as cholecalciferol, 48 IU vitamin E as dl-α-tocopherol acetate, 3 mg vitamin K as menadione, 18 mg pantothenic acid, 6 mg riboflavin, 600 mg choline, 2.4 mg folic acid, 30 mg niacin, 18 mg thiamine, 1.8 mg pyridoxine, 0.03 mg vitamin B_12_, 0.24 mg biotin, 1,200 mg Ca from CaCO_3_, 18 mg Cu from CuSO_4_∙5H_2_O, 120 mg Fe from FeSO_4_, 24 mg Mn from MnSO_4_, 126 mg Zn from ZnSO_4_, 0.36 mg Se from Na_2_SeO_3_, and 0.6 mg I from KI (DSM Nutritional Products Canada Inc., Ayr, ON, Canada).

Grand Valley Fortifiers (Cambridge, ON, Canada)

Calculated using the NRC (2012) ingredient values.

Standardized ileal digestibility.

Standardized total tract digestibility.

The positive control diet (**PC**) contained multiple sources of animal-based proteins (i.e. whey and blood products) and purified plant proteins (i.e. soybean isolate) and was formulated with low DON-contaminated corn (i.e. high complexity). Soybean meal was the main source of protein for all low-complexity (**LC**) diets, with no animal-based protein sources included. Therefore, ‘complexity’ refers to the number of ingredients used in the diet versus describing the matrix of individual ingredients. Of the four LC diets, one was formulated with low DON contaminated corn and without feed additives (negative control; **NC**). The remaining three LC diets were formulated with corn naturally contaminated with 5.6 ppm DON (data not shown). Of the three DON-contaminated diets, one was not supplemented with an additive (**DON‐**), one included a feed additive containing a blend of immune-modulating components [**DON+**; included in complete feed at 2 g/kg; the feed additive blend contained per kg: vitamins (vitamin D3: min. 39,650 I.U.; vitamin E: min. 2,600 I.U.; niacin: min. 1,900 mg; thiamine: min. 440 mg; riboflavin: min. 330 mg; calcium d-pantothenate: min. 1,000 mg; pyridoxine: 220 mg; biotin: 1,000 µg; vitamin B12: 2,000 µg; menadione: min. 80 mg), yeast product (dehydrated yeast autolysate), and an inorganic adsorbent (montmorillonite clay); NutraMix™, Canadian Bio-Systems Inc., Calgary, AB, Canada], and one included fish oil (**DONω3**; 2.5% corn oil substituted with 2.5% fish oil). On days 15, 16, and 17, fresh fecal samples were collected from each pen to determine ATTD of organic matter (**OM**) and energy; fecal samples were frozen at -20°C until further processing.

### Assessment of Acute-Phase Immune Response and Small Intestinal Morphology

On study day 22, 120 pigs were administered an intramuscular injection: two pigs per pen (1 male and 1 female, randomly selected) received 30 μg of *Escherichia coli* LPS per kg BW in 1 mL of saline (strain O55:B5; Sigma-Aldrich Co., St. Louis, MO) and 1 pig per pen (male or female, each from half of the pens) received 1 mL of saline (Huber et al., 2018). A total of 16 pigs were included per treatment for LPS, and 8 pigs per treatment were included per treatment for saline; *n* = 8 experimental units per dietary treatment. Rectal temperature was measured for each pig at 0, 1, 2, and 3 h post-injection. At 3 h, blood samples were collected via suborbital-sinus puncture into plasma vacutainer tubes containing an anticoagulant (EDTA; BD Vacutainer^®^, BD, Franklin Lakes, NJ; Lee et al., 2019). Blood samples were stored on ice for up to 4 h and were centrifuged for 20 min at 3000 × *g* and 4 °C. Plasma was aliquoted into microcentrifuge tubes and stored at ‐20 °C until further analysis. Within 30 min following the final rectal temperature measurement (~ 3.5 h post-injection), pigs were euthanized with an IV injection of 3 mL of pentobarbitol (250 mg/mL; Euthasol, Virbac, TX). Immediately thereafter, the entire intestinal tract was excised, and full gut weight and empty organ weights were measured.

Five-centimeter sections of the jejunum (1.5 m past the ligament of Trietz) and the ileum (0.5 m proximal to the ileo-cecal junction) were removed, rinsed with saline, and placed in 10% formalin until further processing. Tissue samples were prepared according to Carleton et al. (1980) for histological analysis. A Leica DMR fluorescence microscope (Leica Microsystems Inc., Wetzlar, Germany) and Openlab Computer Imaging System (Perkin Elmer, Waltham, MA) was used to measure villi height and crypt depth for the five longest villi per intestinal section.

A commercially available porcine multiplex cytokine panel (granulocyte-macrophage colony-stimulating factor: **GM-CSF**; interlekin-1 α: **IL-1α**; interleukin-1 β: **IL-1β**; interleukin-1 receptor antagonist: **IL-1RA**; interleukin-2: **IL-2**; interleukin-4: **IL-4**; interleukin-6: **IL-6**; interleukin-8: **IL-8**; interleukin-10: **IL-10**; interleukin-12: **IL-12**; interleukin-18: **IL-18**; interferon-γ: **IFN-γ**; tumor necrosis factor α: **TFNα**) was used to analyze plasma cytokines 3 h after LPS or saline injections (Milliplex Map Kit Porcine cytokine/chemokine magnetic bead panel, EMD Millipore Corp., Billerica, MA) as described by Lee et al. (2019). The inter-assay CV was 5.7%.

### Nutrient Analyses

Fecal samples were freeze-dried and finely ground prior nutrient analyses. Experimental diets and fecal samples were analyzed for dry matter (**DM**) and ash contents according to AOAC methods (AOAC, 2005; Methods 930.15 and 942.05, respectively); diets were analyzed in triplicate and fecal samples were analyzed in duplicate. Diet and fecal samples were analyzed for gross energy (**GE**) using a bomb calorimeter (IKA Calorimeter System C 5000; IKA Works Inc., Wilmington, NC) with benzoic acid as the calibration standard. Titanium contents in feed (in triplicate) and fecal (in duplicate) samples were measured as described by Myers et al. (2004) with minor adaptations (24 h digestion at 120 °C in 10 mL tubes and H_2_O_2_ added after precipitate settled in 100 mL volumetric flask), with absorbance of standards and samples measured at 408 nm by spectrophotometry. The acceptable coefficient of variation among replicates was 5% for all analyses. Diets were analyzed by SGS Agrifood Canada (Guelph, ON, Canada) for DM (AOAC, 2005; Method 930.15), crude protein (LECO-FP 428; LECO Instruments Ltd., Mississauga, ON, Canada; AOAC, 2005; Method 968.06), calcium, phosphorus, potassium, and magnesium (AOAC, 2005; Method 985.01). Plant-derived ingredients (data not shown) and diets were analyzed for mycotoxin content by Canadian Bio-Systems Inc. (Calgary, AB, Canada) using Agilent 1100 Series HPLC system (Agilent, Santa Clara, CA) and AB SCIEX 4000 MS/MS (SCIEX, Framingham, MA).

### Calculations and Statistical Analysis

The ATTD of OM and GE were calculated between days 15 and 17 days after weaning (*n* = 8; pen as experimental unit) using the index method as described by Columbus et al. (2010). Digestible energy was calculated by subtracting GE in fecal samples from GE in feed. The SAS (Version 9.4; SAS Inst. Inc., Cary, NC) GLIMMIX procedure was used to analyze all data. Growth performance and ATTD data were analyzed with dietary treatment as the main effect. The LPS challenge data (i.e. rectal temperature, organ weights, small intestinal histomorphology, plasma cytokine concentrations) were analyzed with the main effects of dietary treatment and injection type (i.e. saline or LPS) and their interactions; the rectal temperature model included time and its interactions with dietary treatment and injection type as main effects. For plasma cytokine concentrations (except for INF-γ and IL1-RA), the diet*LPS interaction was removed from the model since it was not significant. Plasma IL-8 and IL-18 concentrations data were log-normal transformed prior to analysis; data were back transformed to present results. Block was included as a random effect in all analyses, repeated measures were considered, and pen was used as the experimental unit. Mean comparisons were conducted using Tukey-Kramer test to separate means when the interaction between diet and injection type was significant. A probability (***P***) of less than 0.05 was considered significant, whereas 0.05 ≤ *P* ≤ 0.10 was considered a tendency, and *P* > 0.10 was considered not significant.

## RESULTS

### Diets

Experimental diet chemical analyses were similar to the calculated values, with some exceptions. The CP content was ~ 13% greater than expected for the PC phase I diet, calcium contents were ~ 27% (average) lower than expected for the PC phase I, NC phases I and II, DON+ phase II, and DONω3 phases I and II diets, and phosphorus contents were ~ 22% (average) lower than expected for PC phase I, NC phase II, DON+ phase II, and DONω3 phases I and II diets (Tables 1 and 2). For all diets, fumonisin, and aflatoxin were below detection limits. The mycotoxin contaminations of the PC and NC diets were below the recommended thresholds for all mycotoxins analyzed; in the phase II PC diet, zearalenone was relatively greater (0.21 ppm) versus all other PC and NC phases (average 0.08 ppm) but still below recommended threshold. The diets formulated with DON-contaminated corn contained ~ 3.5 ppm DON, except for the DON+ phase I diet that contained 4.3 ppm DON. Since all DON diets were made with the same ingredients, and since all other diets had similar DON contamination, it is likely the difference was due to sampling error. In addition, the diets formulated with DON-contaminated corn had relatively greater zearalenone (average 0.34 ppm) versus non-DON-contaminated diets (0.12 ppm), but all were below the recommended limit of 1 ppm for nursery pigs. The common diet (phase III) contained < 1 ppm DON and < 0.20 ppm zearalenone.

**Table 2.** Analyzed nutrient composition and mycotoxin content of experimental diets (as-fed basis)^1^

Item	PC		NC		DON‐		DON+		DONω3		
	Phase I	Phase II	Phase I	Phase II	Phase I	Phase II	Phase I	Phase II	Phase I	Phase II	Phase III
Analyzed nutrient composition, %											
Dry matter	89.74	88.75	89.64	89.02	89.29	88.73	89.82	88.39	89.87	88.63	89.06
Crude protein	26.24	20.63	23.42	23.07	22.89	24.00	22.44	22.48	22.48	22.67	20.87
Calcium	0.70	0.82	0.80	0.65	0.95	0.84	0.93	0.76	0.77	0.73	0.83
Phosphorus	0.65	0.63	0.69	0.61	0.70	0.64	0.72	0.61	0.56	0.58	0.59
Sodium	0.26	0.29	0.25	0.22	0.26	0.26	0.23	0.24	0.26	0.27	0.23
Potassium	1.05	0.76	0.94	0.84	1.05	1.05	0.96	0.99	0.98	0.99	0.83
Magnesium	0.15	0.14	0.16	0.14	0.17	0.17	0.16	0.16	0.15	0.16	0.16
Mycotoxin content, ppm											
Deoxynivalenol	0.60	0.88	0.63	0.70	3.42	3.74	4.29	3.64	3.48	3.04	0.97
Zearalenone	0.08	0.21	0.08	0.09	0.20	0.35	0.48	0.32	0.32	0.37	0.17
Fumonisin	<0.10	<0.10	<0.10	<0.10	<0.10	<0.10	<0.10	<0.10	<0.10	<0.10	<0.10
Aflatoxin	<0.001	<0.001	<0.001	<0.001	<0.001	<0.001	<0.001	<0.001	<0.001	<0.001	<0.001

Experimental diets: [1] complex diet containing multiple sources of plant and animal proteins (positive control; PC), [2] low-complexity (LC) diets containing plant proteins as the main sources of protein, using corn with minimal deoxynivalenol content (negative control; NC), or corn with high DON contents and no feed additive (DON–), or with the feed additive (DON+), or supplemented with fish oil (DONω3). Phases I and II were fed between days 0 and 7 and 8 and 22 after weaning (treatment phases), respectively, and a common phase III was fed to all pigs between days 23 and 42 after weaning (recovery phase).

### Growth Performance and Apparent Total Tract Digestibility

In phase I, ADG, ADFI, and G:F for pigs fed PC and NC were not different and pigs fed DON+ and DONω3 had lower ADG, ADFI, and G:F compared to PC and NC (*P* < 0.05; Table 3). In phase I, pigs fed DON‐ had lower ADG and ADFI than pigs fed PC (*P* < 0.05) and tended to have lower ADFI than pigs fed NC (*P* = 0.054). In phase II, pigs fed DON‐ and DONω3 had lower ADG than pigs fed NC (*P* < 0.05), tended to have lower ADG than pigs fed PC (*P* = 0.073 and *P* = 0.097, respectively), and had lower ADFI and greater G:F than pigs fed PC (*P* < 0.05); intermediate values were observed for pigs fed NC and DON+ for ADFI and G:F. By the end of phase II (end of treatment phase), pigs fed DONω3 tended to have lower BW compared to pigs fed PC and NC (*P* = 0.084 and *P* = 0.079, respectively), which were not different from one another; pigs fed DON‐ and DON+ had intermediate BW at the end of phase II. In phase III and over the entire nursery period, dietary treatment did not affect ADG, ADFI, or G:F. There were no differences in final BW among dietary treatments.

**Table 3.** Effect of nursery diets formulated with deoxynivalenol-contaminated corn and a feed additive or fish oil supplementation on pig growth performance during the nursery period

Item	Dietary treatments^1^					SEM^2^	*P*-value^3^
	PC	NC	DON				
			–	+	ω3		
No.^4^	8	8	8	8	8		
Initial BW, kg	6.7	6.7	6.7	6.7	6.7	0.3	-
Day 22 BW, kg^5^	13.4^x^	13.4^x^	12.7^x,y^	12.9^x,y^	12.6^y^	0.4	0.018
Final BW, kg	26.5	26.1	25.4	26.1	26.3	0.7	0.523
ADG, g							
Phase I	68^a^	53^a,b^	33^b,c^	20^c^	17^c^	12	<0.001
Phase II	406^a,b,x^	410^a^	374^b,y^	392^a,b^	376^b,y^	11	0.006
Phase III	668	664	655	679	698	22	0.232
Overall	472	464	445	462	465	12	0.384
ADFI, g							
Phase I	162^a^	145^a,b,x^	122^b,c,y^	115^c^	107^c^	9	<0.001
Phase II	519^a^	500^a,b^	451^b^	479^a,b^	453^b^	16	0.005
Phase III	1065	1046	1023	1089	1076	43	0.527
Overall	639	620	584	624	605	22	0.167
G:F							
Phase I	0.41^a^	0.35^a^	0.27^a,b^	0.14^b^	0.14^b^	0.09	<0.001
Phase II	0.78^b^	0.82^a,b^	0.83^a^	0.82^a,b^	0.83^a^	0.01	0.027
Phase III	0.63	0.64	0.64	0.62	0.65	0.01	0.291
Overall	0.74	0.75	0.76	0.74	0.77	0.01	0.207
ATTD^6^, %							
OM	85.5^a,b,x^	83.4^c^	84.9^a,b^	84.1^b,c,y^	85.5^a^	0.5	<0.001
GE	82.7^a^	80.3^b^	81.7^a,b^	80.9^b^	82.7^a^	0.6	<0.001
DE, kcal/kg	3809^a^	3698^b^	3746^a,b^	3684^b^	3792^a^	27	<0.001

Experimental diets: [1] complex diet containing multiple sources of plant and animal proteins (positive control; PC), [2] low-complexity diets containing plant proteins as the main sources of protein, using corn with minimal deoxynivalenol content (negative control; NC), or corn with high DON contents and no feed additive (DON‐), or with the feed additive (DON+), or supplemented with fish oil (DONω3). Phases I and II were fed between days 0 and 7 and 8 and 22 after weaning (treatment phases), respectively, and a common phase III was fed to all pigs between days 23 and 42 after weaning (recovery phase).

Standard error of the means.

Main effect *P*-values.

Number of experimental units (pens).

Body weight after the treatment phase (end of phase II).

Apparent total tract digestibility determined between days 15 and 17 after weaning (end of phase II).

Values with different letters within the same row differ (*P* < 0.05).

Values with different letters within the same row tend to differ (*P* ≤ 0.10).

During phase II, pigs fed DONω3 had greater ATTD for OM than pigs fed DON+ and NC (*P* < 0.05; Table 3), pigs fed PC had greater OM digestibility than pigs fed NC and tended to have greater OM digestibility than pigs fed DON+ (*P* < 0.05 and *P* = 0.068, respectively), and pigs fed DON‐ had greater OM digestibility than pigs fed NC (*P* < 0.05). The ATTD of GE and calculated DE were greater for pigs fed DONω3 and PC versus pigs fed NC and DON+ (*P* < 0.05), while intermediate values were observed for pigs fed DON‐.

### LPS-Induced Acute-Phase Response, Relative Organ Weights, and Small Intestinal Histomorphology

At time 0, there were no effects of dietary treatment on rectal temperature (baseline; Table 4). Beginning 1 h after injection, LPS-injected pigs had greater rectal temperature compared to saline-injected pigs, regardless of dietary treatment (*P* < 0.001); dietary treatment did not affect the magnitude of the response.

**Table 4.** Effect of nursery diets formulated with deoxynivalenol-contaminated corn and a feed additive or fish oil supplementation on rectal temperature of pigs that received LPS or saline on day 22 after weaning

Item	Dietary treatments^1^					SEM^4^	LPS challenge^2^		SEM5	*P-*value^3^		
	PC	NC	DON									
			–	+	ω3		–	+		Diet	LPS	Diet * LPS
No.6	16	16	16	16	16		40	40				
Hour after challenge												
0 h	39.9	39.8	39.7	39.8	39.6	0.2	39.8	39.7	0.1	0.798	0.299	0.369
1 h	40.4	40.2	40.2	40.2	40.0	0.2	39.8	40.6	0.1	0.669	<0.001	0.114
2 h	40.5	40.4	40.3	40.5	40.2	0.2	39.9	40.9	0.1	0.615	<0.001	0.533
3 h	40.8	40.3	40.2	40.4	40.1	0.2	39.9	40.8	0.1	0.185	<0.001	0.664

Experimental diets: [1] complex diet containing multiple sources of plant and animal proteins (positive control; PC), [2] low-complexity diets containing plant proteins as the main sources of protein, using corn with minimal deoxynivalenol content (negative control; NC), or corn with high DON contents and no feed additive (DON‐), or with the feed additive (DON+), or supplemented with fish oil (DONω3). Phases I and II were fed between days 0 and 7 and 8 and 22 after weaning (treatment phases), respectively, and a common phase III was fed to all pigs between days 23 and 42 after weaning (recovery phase).

LSmeans for the main effect of LPS challenge.

Main effect *P*-values [dietary treatment, LPS injection, and the interaction between dietary treatment and ^LPS injection].^

Standard error of the means for dietary treatment.

Standard error of the means for LPS challenge.

Number of experimental units. One pig per pen received saline and two pigs per pen received LPS; results were averaged for pigs that received LPS within a pen.

Relative full gut weight was influenced by the interaction between dietary treatment and LPS injection (*P* < 0.05) such that the LPS injection reduced relative full gut weight for NC- and DONω3-fed pigs (*P* < 0.05), while relative full gut weights were not different between LPS- and saline-injected pigs for pigs fed PC, DON‐, or DON+ (Table 5). The LPS injection tended to reduce relative empty gut weights, regardless of dietary treatment (*P* = 0.084). Relative liver weight tended to be influenced by the interaction between dietary treatment and LPS injection (*P* = 0.075) such that the LPS injection increased relative liver weight only for pigs fed DON+ (*P* < 0.05). Relative small intestine weight tended to be influenced by the interaction between dietary treatment and LPS injection (*P* = 0.052) such that the LPS injection reduced relative small intestine weight only for pigs fed NC (*P* < 0.05). Pigs fed DON+ and DONω3 tended to have greater relative large intestine weights than pigs fed PC, regardless of LPS injection (*P* = 0.085 and *P* = 0.074, respectively), and LPS injection tended to decrease relative large intestine weight, regardless of dietary treatment (*P* = 0.091). Relative spleen and stomach weights were not influenced by the main effects of dietary treatment, LPS injection, or the interaction between dietary treatment and LPS injection.

**Table 5.** Effect of nursery diets formulated with deoxynivalenol-contaminated corn and a feed additive or fish oil supplementation on physical characteristics of the gastrointestinal tract for pigs that received LPS or saline on day 22 after weaning

Item	Dietary treatments^1^										SEM^3^	*P-*value^2^		
	PC		NC		DON									
					–		+		ω3					
	S	LPS	S	LPS	S	LPS	S	LPS	S	LPS		Diet	LPS	Diet * LPS
No.^4^	8	8	8	8	8	8	8	8	8	8				
Organ weight, g/kg BW														
Full gut	148^a,b,c^	134^b,c^	178^a^	123^c^	151^a,b,c^	131^c^	151^a,b,c^	134^b,c^	163^a,b^	127^c^	7	0.446	<0.001	0.018
Empty gut	88.1	88.8	98.3	83.8	89.8	89.6	89.4	89.4	95.4	89.6	3.9	0.561	0.084	0.170
Spleen	3.84	4.01	3.71	3.97	3.88	3.96	4.02	3.63	4.52	4.12	0.41	0.771	0.770	0.721
Liver	32.9^a,b^	35.2^a,b^	36.5^a,b^	35.0^a,b^	34.5^a,b^	37.5^a,b^	31.5^b^	39.1^a^	36.4^a,b^	38.9^a^	1.7	0.092	0.005	0.075
Stomach	7.72	7.29	7.46	7.18	7.64	7.66	7.18	8.25	7.73	7.98	0.43	0.573	0.580	0.260
Small intestine	60.4^a,b^	62.4^a,b^	70.0^a^	57.8^b^	61.9^a,b^	61.5^a,b^	60.6^a,b^	61.6^a,b^	65.7^a,b^	62.1^a,b^	2.9	0.591	0.111	0.052
Large intestine	17.7	19.2	20.8	18.8	20.3	20.5	21.6	19.9	22.0	19.5	1.0	0.059	0.091	0.122
Jejunal morphology, µm														
Villus height	825	678	812	655	797	665	790	818	728	630	47	0.040	<0.001	0.176
Crypt depth	269^c^	316^a,b,c^	284^b,c^	343^a,b,c^	292^b,c,y^	357^a,b,x^	274^c^	384^a^	329^a,b,c^	328^a,b,c^	20	0.257	<0.001	0.011
Villus:crypt ratio	3.11	2.19	2.74	1.92	2.90	1.91	2.93	1.93	2.29	1.97	0.24	0.126	<0.001	0.349
Ileal morphology, µm														
Villus height	805	681	726	694	700	666	820	690	643	608	42	0.002	<0.001	0.222
Crypt depth	319^a,b^	265^a,b^	340^a,x^	263^a,b,y^	302^a,b^	283^a,b^	252^a,b^	261^b^	323^a,b^	269^a,b^	20	0.041	<0.001	0.084
Villus:crypt ratio	2.55^a,b^	2.68^a,b^	2.12^b^	2.70^a,b^	2.35^a,b^	2.42^a,b^	3.28^a^	2.69^a,b^	2.08^b^	2.29^b^	0.23	0.005	0.492	0.057

Experimental diets: [1] complex diet containing multiple sources of plant and animal proteins (positive control; PC), [2] low-complexity diets containing plant proteins as the main sources of protein, using corn with minimal deoxynivalenol content (negative control; NC), or corn with high DON contents and no feed additive (DON–), or with the feed additive (DON+), or supplemented with fish oil (DONω3). Phases I and II were fed between days 0 and 7 and 8 and 22 after weaning (treatment phases), respectively, and a common phase III was fed to all pigs between days 23 and 42 after weaning (recovery phase).

Main effect *P*-values [dietary treatment, LPS injection, and the interaction between dietary treatment and ^LPS injection].^

Standard error of the means.

Number of experimental units. One pig per pen received saline and two pigs per pen received LPS; results were averaged for pigs that received LPS within a pen.

Values with different letters within the same row differ (*P* < 0.05).

Values with different letters within the same row tend to differ (*P* ≤ 0.10).

The LPS injection reduced jejunal villus height and the jejunal villus height-to-crypt depth ratio (*P* < 0.001; Table 5). Pigs fed DON+ had longer jejunal villi than pigs fed DONω3, regardless of LPS injection (*P* < 0.05), while pigs fed PC, NC, and DON‐ had intermediate jejunal villus heights. Jejunal crypt depth was influenced by the interaction between dietary treatment and LPS injection (*P* < 0.05) such that the LPS injection (versus saline) increased jejunal crypt depth only for pigs fed DON‐ (tendency; *P* = 0.061) and DON+ (*P* < 0.05). The LPS injection reduced ileal villus height (*P* < 0.001). Pigs fed PC and DON+ had greater ileal villi heights than DONω3-fed pigs, regardless of LPS (versus saline) injection (*P* < 0.05), while pigs fed NC and DON‐ had intermediate villus heights. Ileal crypt depth tended to be influenced by the interaction between dietary treatment and LPS injection (*P* = 0.084), where LPS injection tended to reduce ileal crypt depth only for pigs fed NC in comparison to saline-injected animals (*P* = 0.064). The villus height-to-crypt depth ratio also tended to be influenced by the interaction of dietary treatment and LPS injection (*P* = 0.057).

**Figure 1.**
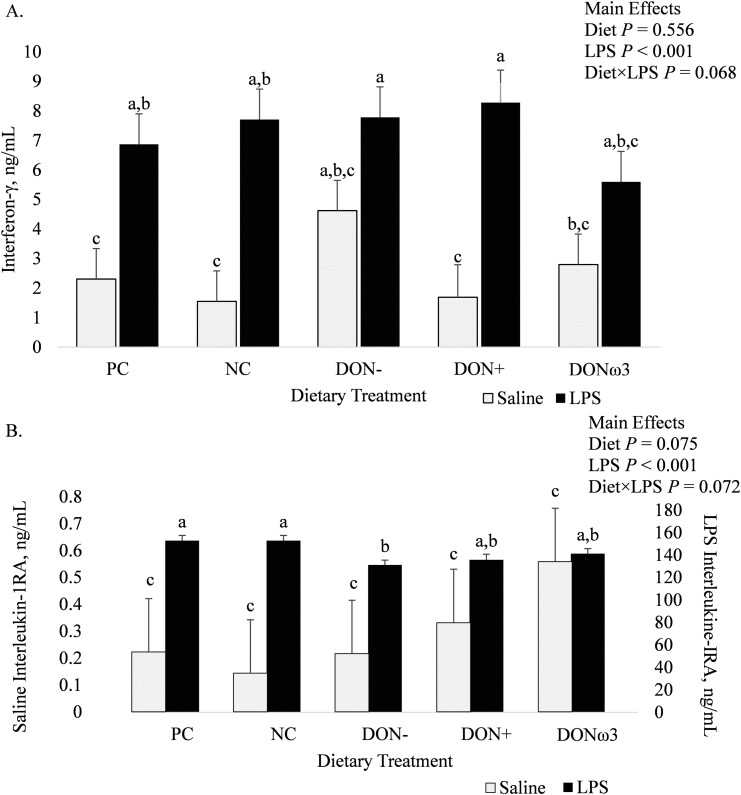
Effect of nursery diets formulated with deoxynivalenol-contaminated corn and a feed additive or fish oil supplementation on plasma concentrations of (**A**) IFN-γ and (**B**) IL-1RA for pigs 3 h after receiving lipopolysaccharide (LPS) or saline on day 22 after weaning. Experimental diets: [1] complex diet containing multiple sources of plant and animal proteins (positive control; PC), [2] low-complexity diets containing plant proteins as the main sources of protein, using corn with minimal deoxynivalenol content (negative control; NC), or corn with high DON contents and no feed additive (DON‐), or with the feed additive (DON+), or supplemented with fish oil (DONω3). Phases I and II were fed between days 0 and 7 and 8 and 22 after weaning (treatment phases), respectively. (**B**) Left *y*-axis represents saline values and right *y*-axis represents LPS values. *n =* 8. ^a–c^Values with different letters differ (*P* < 0.05).

Of the plasma cytokines tested, only interferon-γ and interleukin-1 receptor antagonist concentrations tended to be influenced by the interaction between dietary treatment and LPS injection (*P* = 0.068 and 0.072, respectively; Figure 1 A and B, respectively). The LPS injection increased plasma IFN-γ concentrations in comparison to saline injection for pigs fed PC, NC, and DON+ (*P* < 0.05), but did not influence plasma IFN-γ concentrations for pigs fed DON‐ and DONω3 (Figure 1 A). There were no differences in plasma IL-1RA concentrations among dietary treatments for saline-injected pigs, but for LPS-injected pigs, plasma IL-1RA concentrations were greater for pigs fed PC and NC compared to pigs fed DON‐ (*P* < 0.05), while intermediate values were observed for LPS-injected pigs fed DON+ and DONω3 (Figure 1 B). The remaining plasma cytokines were not influenced by dietary treatment but LPS injection increased the concentration of all analyzed plasma cytokines (*P* < 0.001; Table 6).

**Table 6.** Effect of nursery diets formulated with deoxynivalenol-contaminated corn and a feed additive or fish oil supplementation on plasma cytokine concentrations (ng/mL) for pigs 3 h after receiving LPS or saline injections on day 22 after weaning

Item	Dietary treatments^1^					SEM^4^	LPS challenge^2^		SEM5	*P-*value^3^	
			DON								
	PC	NC	–	+	ω3		–	+		Diet	LPS
No.^6^	16	16	16	16	16		40	40			
GM-CSF	0.03	0.02	0.03	0.03	0.02	0.005	0.01	0.04	0.004	0.950	<0.001
IL-1α	0.04	0.04	0.05	0.07	0.06	0.02	0.01	0.09	0.01	0.599	<0.001
IL-1β	2.09	2.54	2.56	2.81	3.74	0.53	0.18	5.31	0.33	0.265	<0.001
IL-2	0.13	0.12	0.16	0.27	0.13	0.06	0.06	0.26	0.03	0.335	<0.001
IL-4	0.20	0.32	0.26	0.96	0.19	0.30	0.25	0.52	0.14	0.320	<0.001
IL-6	2.98	2.64	2.50	2.58	2.95	0.55	0.04	5.42	0.35	0.955	<0.001
IL-8	0.41	0.64	0.66	0.89	0.76	0.47	0.03	13.38	4.04	0.873	<0.001
IL-10	0.26	0.28	0.29	0.56	0.25	0.11	0.14	0.52	0.05	0.226	<0.001
IL-12	2.02	1.54	2.03	1.64	1.93	0.25	0.84	2.83	0.16	0.523	<0.001
IL-18	1.17	0.98	1.09	1.41	1.28	0.23	0.65	2.13	0.19	0.583	<0.001
TNFα	0.80	0.90	0.97	0.84	1.18	0.24	0.02	1.86	0.15	0.798	<0.001

Experimental diets: [1] complex diet containing multiple sources of plant and animal proteins (positive control; PC), [2] low-complexity diets containing plant proteins as the main sources of protein, using corn with minimal deoxynivalenol content (negative control; NC), or corn with high DON contents and no feed additive (DON–), or with the feed additive (DON+), or supplemented with fish oil (DONω3). Phases I and II were fed between days 0 and 7 and 8 and 22 after weaning (treatment phases).

LSmeans for the main effect of LPS challenge.

Main effect *P*-values (dietary treatment and LPS injection; the interaction between dietary treatment and LPS injection was not significant for any cytokine therefore, only main effects are presented).

Standard error of the means for dietary treatment.

Standard error of the means for LPS challenge.

Number of experimental units. One pig per pen received saline and two pigs per pen received LPS; results were averaged for pigs that received LPS within a pen.

## DISCUSSION

The objectives of the current study were to evaluate the effects of DON-contaminated low-complexity nursery diets containing either an immune-modulating feed additive or fish oil on growth performance, immune response to an *E. coli* LPS challenge, gut morphology, and component digestibility. During the treatment phase (nursery phases I and II), diet complexity did not affect growth performance (ADG, ADFI, or G:F) or small intestinal morphology, which resulted in BW not being different at the end of phase II (study day 22) for pigs fed the high (PC)- or low (NC)-complexity diets with no DON contamination, even though low-complexity diets had poorer OM and energy digestibilities. Likely, growth performance, and small intestinal morphology were not different for pigs fed NC versus PC since feed intake was not affected by diet complexity. These results are in contrast with others that demonstrated reduced feed intake for pigs fed low-complexity diets immediately after weaning (Huber et al., 2018; Lariviere et al., 2021), however, in all cases, there were no differences in BW by the end of the nursery period. Therefore, feeding low-complexity nursery diets could be a means to reduce feed costs, without negatively impacting overall nursery growth performance.

Diets contaminated with > 3.5 ppm DON have been linked to reduced feed intake and weight gain in nursery pigs (Van Le Thanh et al., 2015). Indeed, in the current study, ADFI and ADG were compromised during phases I and II for pigs that received DON-contaminated diets with no feed additive versus pigs that received PC. However, in phase II, pigs fed the DON‐ diet had improved G:F versus pigs that received PC, which could be the result of gradual acclimatization to DON, as Wellington et al. (2020) noted the strength of the negative linear relationship between DON contamination and ADG decreased over time for finisher pigs. Moreover, it has also been suggested that contamination of grain by fungi may improve nutrient digestibility due to greater endogenous amylolytic and proteolytic activities in the kernel at harvest (Matthäus et al., 2004; Kong et al., 2015). The ATTD of OM was greater for pigs fed the low-complexity diet contaminated with DON (DON‐) versus the identical low-complexity diet formulated with clean corn (NC), which could also contribute to improved feed efficiency.

Following the recovery period (phase III), all pigs achieved a similar body weight. Therefore, pigs with suppressed growth during phases I and II were able to achieve complete compensatory growth when fed a common diet for 20 days. Others also demonstrated complete compensatory growth in grower pigs after a 2-week DON-contaminated feeding period followed by a 3-week recovery period (Dänicke et al., 2004) and in nursery pigs only when feeding low levels of DON (~ 1.5 ppm; Larivière et al., 2021), while DON contamination of ~ 3.5 ppm in wheat- and soybean meal- based diets had no long-term impact on nursery pig growth performance (Pasternak et al., 2018). Conversely, when a short-term acute DON challenge (3 ppm for 7 days) was applied in the finisher period, pigs were not able to recover overall ADFI or ADG (Serviento et al., 2018). Therefore, although DON contamination impaired performance early in the nursery period, compensatory growth may rescue these losses if nursery pigs are given the opportunity to consume non-contaminated diets.

In the current study, pigs fed DON+ had no growth performance advantage over those fed DON‐ indicating the feed additive did not rescue growth performance for nursery pigs fed low-complexity, DON-contaminated diets. Others have demonstrated that blends of antioxidants, absorbents, yeast cell wall, and/or organic acids improved growth performance when pigs were fed diets with ~ 3 ppm DON (Van Le Thanh et al., 2015; Jin et al., 2017). Moreover, in the current study, feeding low-complexity, DON-contaminated diets with no feed additive (DON‐) and the addition of fish oil (DONω3) reduced ADFI and ADG versus pigs fed the high complexity (PC) diet. Though a more drastic reduction in feed intake was noted when fish oil was included at levels greater than 2.5% (Huber et al., 2018), it is likely that both DON contamination and fish oil negatively affect diet palatability. Finally, the feed additive provided some benefit for jejunal and ileal villus heights (versus DONω3), even though DON contamination between 1.5 and 3 ppm has been linked to reduced jejunal villus height (Bracarense et al., 2012; Liu et al., 2020). Although, it should be noted that pigs fed DON-contaminated diets with no feed additive (i.e. DON‐) did not experience reduced villus height compared to pigs fed an identical diet with no DON contamination (i.e. NC) or to pigs fed the high complexity (i.e. PC) nursery diet. Therefore, neither the immune-modulating feed additive or 2.5% fish oil provided benefits for growth performance when added to low-complexity, DON-contaminated nursery diets, but small intestinal morphology could be enhanced by the immune-modulating feed additive in instances when feed intake is reduced.

In the current study, to assess immune response, acute immune system stimulation was induced with one intramuscular injection of *E. coli* LPS, which was successful as marked by an increase in rectal temperature and plasma *TNF*α (indicative of systemic inflammation; Tran et al., 2018) and reduction in small intestinal villus height. There was no difference in cytokine response to the LPS challenge between pigs fed PC and NC diets. This is in accordance with other studies that found no difference for immune response to LPS due to diet complexity (Dritz et al., 1996; Gaines et al., 2003). Conversely, dietary DON can have both immunosuppressive and immunostimulatory effects on pigs, depending on exposure concentration and duration (Bondy and Pestka, 2000; Cheng et al., 2006; Liu et al., 2020). Though all 13 plasma cytokines measured in the current study were elevated in response to the LPS challenge, only IL-1RA and IFN-γ tended to be influenced by the interaction between diet and injection type (LPS versus saline). During the LPS challenge, pigs fed DON‐ had lower plasma IL-1RA concentrations than PC- and NC-fed pigs, which could be indicative of reduced capacity to mediate inflammation in response to endotoxin-induced injury (Arend et al., 1998) for pigs fed low-complexity, DON-contaminated nursery diets with no immune-modulating feed additives. On the other hand, pigs fed DON‐ and DONω3 experienced no increase in plasma IFN-γ concentration in response to LPS, which could indicate elevated basal plasma IFN-γ concentrations due to DON exposure. In fact, pigs fed DON‐ and DONω3 had 2.6 and 1.6 × greater basal plasma IFN-γ than those fed PC, NC, and DON+. Thus, exposure to DON could increase plasma concentrations of IFN-γ as others have found greater ileal mucosal mRNA expression of IFN-γ for pigs fed diets contaminated with ~ 3.5 ppm DON (Bracarense et al., 2012; Lessard et al., 2015) and reduced serum total antioxidant content for pigs fed diets with 8 ppm DON (Reddy et al., 2018). This suggests DON exposure between 3 and 8 ppm induces systemic and tissue-specific pro-inflammatory states, which negatively affect host immune competence. Conversely, pigs fed DON-contaminated diets with the feed additive (DON+) underwent a similar IFN-γ response to LPS as pigs fed diets without DON contamination (i.e. PC and NC), indicating that the feed additive rescued immune function when pigs were fed DON-contaminated nursery diets.

## CONCLUSION

In summary, pigs fed low-complexity nursery diets formulated with only plant-based proteins for the first 3 weeks after weaning experienced no difference in growth performance, small intestinal morphology, and immune response to an LPS challenge compared to pigs fed high complexity nursery diets. However, inducing ~ 3.5 ppm DON with naturally contaminated corn in low-complexity diets reduced growth performance and altered certain immune and small intestinal morphology parameters during an LPS challenge. The immune-modulating feed additive, but not fish oil supplementation, offered marginal benefits for small intestinal villus height and immune response for pigs fed DON-contaminated, low-complexity nursery diets. Compensatory growth was expressed following a 3-week recovery period resulting in BW not different for all pigs at nursery exit. Therefore, the addition of the feed additive may mediate the immunosuppressive impact of chronic exposure to DON-contaminated, low-complexity nursery diets, but providing non-contaminated diets in nursery phase III was sufficient to rescue nursery exit BW, regardless of DON contamination and feed additive or fish oil inclusion in previous phases.

## Abbreviations

ATTD: apparent total tract digestibility
DM: dry matter
DON: deoxynivalenol
GE: gross energy
HC: high complexity
IFN-γ: interferon-γ
IL-1β: interleukin-1β
IL-1RA: interleukin-1 receptor antagonist
LC: low-complexity
LPS: lipopolysaccharide
NC: negative control
OM: organic matter
P: probability
PC: positive control
SBM: soybean meal
SID: standardized ileal digestibility
STTD: standardized total tract digestibility
TNF-α: tumor necrosis factor α
